# Higher consumption of sulfur microbial diet is associated with the increased risk of chronic kidney disease

**DOI:** 10.3389/fnut.2025.1716058

**Published:** 2026-01-13

**Authors:** Mengxia Li, Haodong Zhao, Yujie Bai, Jingsi Chen, Cailong Chen, Liqiang Qin, Zhengbao Zhu, Jun Liu, Zheng Zhang

**Affiliations:** Children’s Hospital of Soochow University, Department of Nutrition and Food Hygiene, Department of Epidemiology, School of Public Health, Soochow University, Suzhou, China

**Keywords:** chronic kidney disease, mediation analyses, metabolic disorders, substitution analyses, sulfur microbial diet

## Abstract

**Background:**

Emerging evidence suggested that dietary patterns linked to sulfur-metabolizing bacteria in the stool may influence kidney health. We aimed to investigate the association between the sulfur microbial diet and chronic kidney disease (CKD).

**Methods:**

We included 192,282 participants from the UK Biobank. The sulfur microbial diet score was derived from a 24-h dietary assessment, calculated by summing the products of the *β*-coefficients and their corresponding portion sizes. CKD was defined using the International Classification of Diseases, 10th revision codes (ICD-10). Cox proportional hazard models were used to estimate the hazard ratio (HR) and 95% confidence interval (CI). Mediation analyses were employed to examine potential mediators, primarily focusing on indicators of metabolic disorders and systemic inflammation. Substitution analyses were performed to evaluate the impact of replacing one dietary component of the sulfur microbial diet with another.

**Results:**

After a median follow-up of 12.17 years, 4,739 incident CKD cases were identified. The fully adjusted HR of CKD for participants in the highest quartile was 1.15 (95% CI: 1.06, 1.24) compared with those in the lowest quartile. Substituting one serving per day of low-calorie drinks or sweets and desserts with an equal amount of other vegetables was significantly associated with a 7% or 5% reduction in CKD risk. In the mediation analyses, metabolic disorder factors accounted for 1.27–23.13% of the association between the sulfur microbial diet and CKD, while BMI explained 23.13% of the association.

**Conclusion:**

Higher sulfur microbial diet intake was associated with an increased risk of CKD, which was partially mediated by metabolic disorders. Replacing low-calorie drinks or sweets and desserts with other vegetables was related to a significant reduction in CKD risk. These findings highlighted the role of sulfur-metabolizing bacteria in the relationship between diet and CKD.

## Introduction

Chronic kidney disease (CKD), characterized by a reduced glomerular filtration rate (GFR) or elevated albuminuria (1), affects approximately 15–20% of the adult population (2). CKD is associated with high rates of both disability and mortality, significantly impairing patients’ quality of life and imposing a substantial economic burden (3). CKD is often asymptomatic in its early stages, making early diagnosis challenging (4). Adopting a healthy lifestyle may be an effective approach to preventing CKD. As a key component of a healthy lifestyle, the association between diet and CKD has been demonstrated in several studies (5, 6). Thus, identifying the dietary factors associated with CKD development would be beneficial for disease prevention.

The sulfur microbial diet is a dietary pattern associated with sulfur-metabolizing bacteria in stool, which convert dietary sulfur into hydrogen sulfide (H_2_S) (7). Excessive exposure to H_2_S can be harmful to the host, causing damage to the intestinal epithelium and triggering inflammatory responses (8). A higher intake of the sulfur microbial diet was previously reported to be associated with an increased risk of colorectal cancer and obesity (9, 10). More importantly, this dietary pattern has also been linked to an elevated risk of nonalcoholic fatty liver disease (NAFLD) (11). Given that the liver and kidneys are interconnected organs vital for maintaining bodily homeostasis, where damage to one can activate pathogenic pathways that affect the other (12), it was compelling to investigate whether the sulfur microbial diet also modulated the risk of CKD. The bidirectional relationship between kidney function and the gut microbiota, referred to as the “gut-kidney axis,” has aroused increasing interest (13–15). Dysbiosis of the gut microbiota could lead to metabolite disorders that were involved in CKD (16, 17). However, limited research has investigated the link between dietary patterns associated with specific gut microbiota, such as the sulfur microbial diet, and CKD. Poor dietary habits induced gut microbial dysbiosis, which in turn drove metabolic dysregulation and chronic inflammation, thereby increasing the risk of CKD (18, 19). In addition, metabolic disorders, including obesity, dysregulated blood glucose, dyslipidemia, and hypertension, as well as inflammation, were significantly associated with the development and progression of CKD (20, 21). We hypothesized that increased consumption of a sulfur microbial diet may initiate or accelerate CKD, with metabolic disorders or inflammation acting as intermediary factors.

Therefore, we utilized a large-scale prospective cohort to investigate the association between the sulfur microbial diet and the risk of CKD. Furthermore, we conducted mediation analyses to explore potential pathways underlying this association, and performed substitution analyses to evaluate the impact of replacing one dietary component of the sulfur microbial diet with another.

## Methods

### Study population

The UK Biobank was a large prospective population-based cohort of more than 500,000 participants aged 37–73 years, recruited from 22 assessment centers across England, Scotland, and Wales between 2007 and 2010 (22). At a baseline visit, participants completed a face-to-face interview, a touch screen questionnaire, physical examinations, and provided biological samples. All participants provided written informed consent. The UK Biobank had ethical approval from the North West Multi-Centre Research Ethics Committee. These analyses were conducted under the auspices of Application Number 91185.

**Figure 1:**
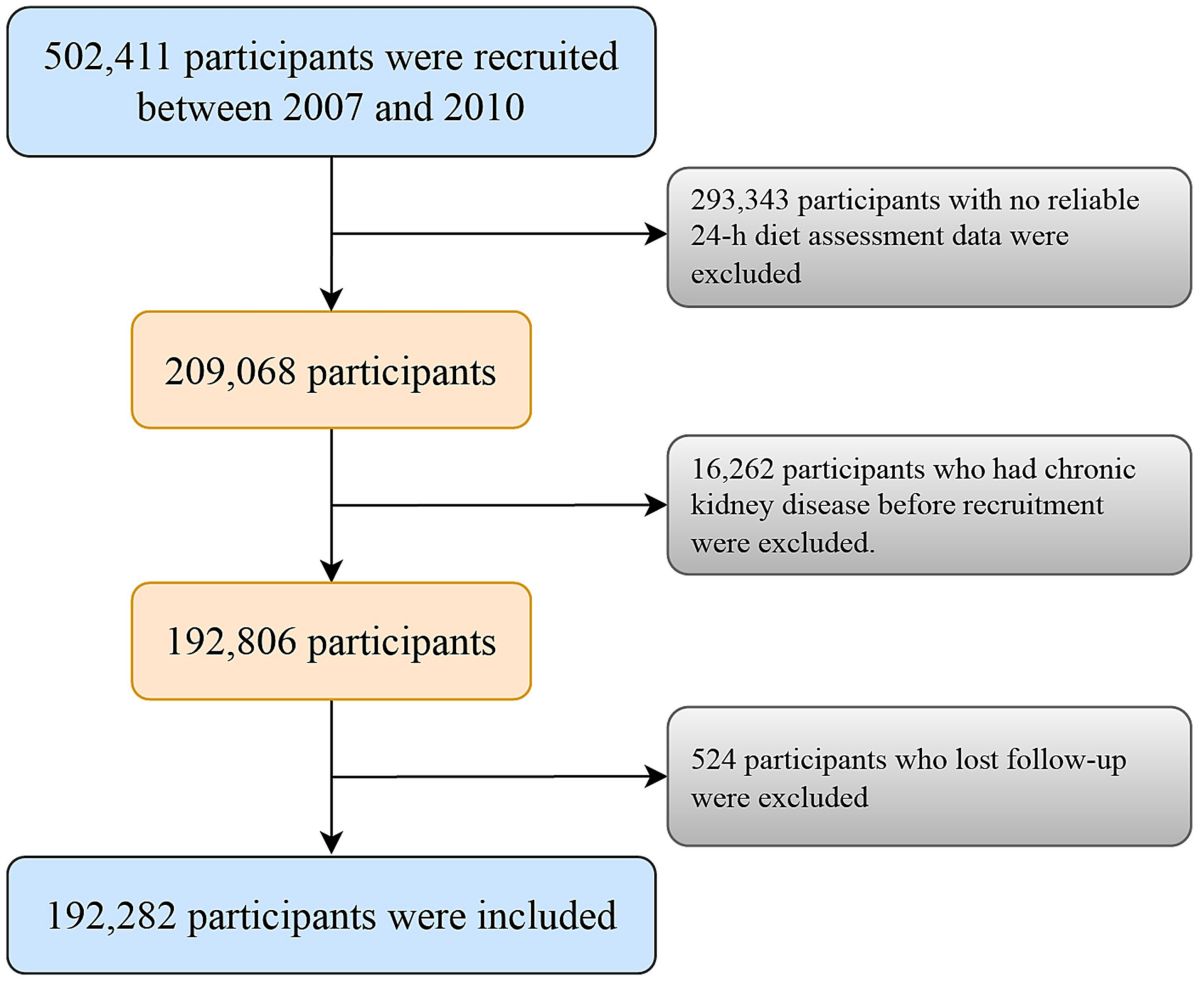
Participant included flow diagram.

Among the 502,411 participants, we excluded those with no reliable 24-h diet assessment data (*n* = 293,343), a history of CKD before recruitment (*n* = 16,262), and those lost to follow-up (*n* = 524). We classified individuals as having unreliable 24-h diet assessments if they did not participate in the dietary assessment (<1 time) or lacked dietary data on sulfur microbial diet. Finally, 192,282 participants were included in the final analyses (Figure 1).

### Sulfur microbial diet score calculation

Dietary information from participants was collected using the Oxford WebQ, a web-based 24-h recall questionnaire (23). Participants in the UK Biobank were invited to complete the Oxford WebQ on five occasions, and we calculated mean values from the available data to provide an accurate representation of their dietary intake. In a previous study, 43 distinct sulfur-metabolizing bacterial species were identified and their associations with dietary intake were explored using reduced rank regression models and stepwise linear regression analyses (7). It was worth noting that the sulfur microbial diet was initially constructed in a cohort of elder men (7), but a subsequent study extended this research by combining the Men’s Lifestyle Validation Study and the Mind Body Study of female registered nurses (9).

The analysis identified seven food groups. Positive associations were observed for processed meats, liquor, and low-calorie drinks, while negative associations were found for beer, fruit juice, legumes, other vegetables, and sweets or desserts. The sulfur microbial diet score was derived by summing of the products of the *β*-coefficients and their corresponding serving sizes (10), with a higher score indicating greater enrichment of sulfur-metabolizing bacteria (Supplementary Table 1).

### Outcome assessment

The outcome of this study was CKD, which was defined using the International Classification of Diseases, 10th revision (ICD-10) codes I12.0, I13.1, I13.2, N18, N18.0, N18.1, N18.2, N18.3, N18.4, N18.5, N18.8, and N18.9 (24). Participants with CKD prior to recruitment are defined based on the ICD-10 code mentioned above, specifically those for whom the first onset date is on or before the recruitment date. A baseline estimated GFR (eGFR) of less than 60 mL/min/1.73 m^2^ was utilized as a supplementary diagnostic criterion for baseline CKD.

Participants were followed from the date of their initial visit to the assessment center until the earliest of the following: first diagnosis of CKD, death, or the end of the follow-up period (September 30, 2021).

### Covariates and mediators

We considered the following baseline factors as covariates: age, sex, race (White, Non-white), body mass index (BMI), Townsend deprivation index, educational qualification (College or University; A levels/AS levels, O levels/GCSEs or equivalent; CSEs, NVQ, HND, HNC or Other professional qualifications; None of the above; or Missing), household income (<£18,000, £18,000–£30,999, £31,000–£51,999, >£52,000, or Missing), employment status [employed, including those in paid employment or self-employed, retired, doing unpaid or voluntary work, or being full or part-time students, and unemployed (those who chose others)], self-reported smoking status (Never, Previous, Current, or Missing), alcohol intake (Never, Previous, ≤3 times a month, once or twice a week, ≥3 times a week, or Missing), diabetes, cardiovascular disease (CVD), hypertension, and energy intake (kJ/day). The International Physical Activity Questionnaire was used to calculate the metabolic equivalent of task (MET), and physical activity levels were classified into three categories: low (<600 MET-min/week), moderate (600–3,000 MET-min/week), and high (>3,000 MET-min/week).

Because metabolic disorders and inflammation may be potential mediators of the association between diet and CKD (20, 25, 26), we considered several factors as possible mediators. These included waist circumference and BMI, calculated using the standard formula: weight (kg)/height squared (m^2^). Blood samples were collected and analyzed for glucose, triglycerides, total cholesterol, HDL cholesterol, and C-reactive protein (CRP) using a Beckman Coulter AU5800. In addition, blood pressure measurements, including both systolic blood pressure (SBP) and diastolic blood pressure (DBP), were also evaluated as potential mediators.

### Statistical analyses

Baseline characteristics across the quartiles of sulfur microbial diet score were presented as means ± standard deviation (SD) for continuous variables and as numbers with percentages (%) for categorical variables. We used Cox proportional hazard regression models to estimate the hazard ratio (HR) and 95% confidence interval (CI) of CKD associated with sulfur microbial diet. The proportional hazard assumption was checked using a global test with Schoenfeld residuals (*p* = 0.179). Model 1 was adjusted for age, sex, and race. Model 2 included further adjustments for BMI, Townsend deprivation index, educational qualification, household income, employment status, smoking status, alcohol intake, physical activity level, and history of CVD, diabetes, and hypertension. Model 3 extended Model 2 by additionally adjusting for energy intake. Unless otherwise specified, results from the fully adjusted model (Model 3) were presented. Furthermore, we employed restricted cubic splines (RCS) to visualize the dose–response relationship between the sulfur microbial diet and the risk of CKD.

Additionally, we performed substitution analyses using a Leave-One-Out model to assess the impact of replacing one dietary component of the sulfur microbial diet with another (27). Mediation analyses were conducted to examine the potential mediating roles of obesity (assessed by BMI and waist circumference), hyperglycemia, dyslipidemia (including triglycerides, total cholesterol, and HDL cholesterol), hypertension, and systemic inflammation (reflected by CRP) in the association between the sulfur microbial diet and CKD risk. These analyses were performed using the PROC CAUSALMED procedure in SAS (28).

To test the robustness of the results, we conducted several sensitivity analyses by excluding those who experienced events within the first two years of follow-up to address potential reverse causality, those who provided 24-h diet recall data only once, those with extreme energy intake (<3,300 or >18,800 kJ for males or <2000 or >14,600 kJ for females), and those who had a history of diabetes or CVD at baseline. In addition, we further adjusted the cancer history, medication use, or a healthy diet score. The healthy diet score was based on the following criteria: fruits: ≥3 servings/day, vegetables: ≥3 servings/day, fish: ≥2 servings/week, processed meats: ≤1 serving/week, unprocessed red meats: ≤1.5 servings/week, whole grains: ≥3 servings/day, refined grains: ≤1.5 servings/day. The healthy diet score ranged from 0 to 7 (29). To mitigate bias from differences in absolute intake levels, we recalculated the sulfur microbial diet score using the Z-standardized portion size. We also accounted for competing risks by treating death prior to a CKD event as a competing event and constructed a competing risks model. Finally, we refined the baseline CKD definition by incorporating a random urine albumin creatinine ratio (UACR) ≥ 3 mg/mmol (30).

We performed stratified analyses according to age (<60, ≥60), sex (male, female), race (white, non-white), alcohol intake (never, <3 times/week, ≥3 times/week), and smoking status (never, current/previous). *p* value for interaction was calculated by including the cross-product terms in the analysis.

All statistical analyses were performed using SAS 9.4 (SAS Institute Inc.) and R (version 4.4.1). All *p* values were reported as two-tailed, and statistical significance was set at *p* < 0.05.

## Results

### Baseline characteristics

The baseline characteristics of the study participants, stratified by sulfur microbial diet score, were presented in Table 1. Participants with higher scores tended to be younger, male, non-white, and have higher household incomes. They were also more likely to be employed, current smokers, and to have lower educational qualification and physical activity levels. Additionally, this group had a higher average BMI despite lower energy intake, a lower prevalence of binge drinking, and a higher prevalence of diabetes and CVD.

**Table 1:** UK Biobank participant characteristics by the sulfur microbial diet score.

Variables	Quartiles of sulfur microbial diet score				
	Total (*n* = 192,282)	*Q*1 (*n* = 48,082)	*Q*2 (*n* = 48,044)	*Q*3 (*n* = 48,082)	*Q*4 (*n* = 48,074)
Age (y, mean ± SD)	55.97 ± 7.93	56.58 ± 7.89	56.44 ± 7.82	55.95 ± 7.88	54.91 ± 8.03
Sex, *n* (%)					
Male	86,440 (44.95)	22,747 (47.31)	20,275 (42.20)	20,531 (42.70)	22,887 (47.61)
Female	105,842 (55.05)	25,335 (52.69)	27,769 (57.80)	27,551 (57.30)	25,187 (52.39)
Race, *n* (%)					
White	184,354 (95.88)	46,156 (95.99)	46,329 (96.43)	46,061 (95.80)	45,808 (95.29)
Non-white	7,928 (4.12)	1,926 (4.01)	1,715 (3.57)	2,021 (4.20)	2,266 (4.71)
BMI (kg/m^2^, mean ± SD)	26.90 ± 4.61	26.71 ± 4.51	26.44 ± 4.41	26.69 ± 4.45	27.76 ± 4.94
TDI (mean ± SD)	−1.59 ± 2.87	−1.56 ± 2.87	−1.69 ± 2.81	−1.64 ± 2.84	−1.47 ± 2.94
Household income, *n* (%)					
Less than 18,000	26,395 (13.73)	7,585 (15.78)	6,264 (13.04)	6,121 (12.73)	6,425 (13.36)
18,000–30,999	41,753 (21.71)	11,095 (23.08)	10,566 (21.99)	10,125 (21.06)	9,967 (20.73)
31,000–51,999	49,402 (25.69)	12,156 (25.28)	12,273 (25.55)	12,479 (25.95)	12,494 (25.99)
Greater than 52,000	55,185 (28.70)	12,201 (25.38)	13,984 (29.11)	14,580 (30.32)	14,420 (30.00)
Missing	19,547 (10.17)	5,045 (10.49)	4,957 (10.32)	4,777 (9.94)	4,768 (9.92)
Educational qualification, *n* (%)					
College or University degree	82,297 (42.80)	19,906 (41.40)	21,972 (45.73)	21,459 (44.63)	18,960 (39.44)
A levels/AS levels, O levels/GCSEs or equivalent	64,988 (33.80)	16,050 (33.38)	15,918 (33.13)	16,160 (33.61)	16,860 (35.07)
CSEs, NVQ, HND, HNC or Other professional qualifications	28,138 (14.63)	7,450 (15.49)	6,462 (13.45)	6,592 (13.71)	7,634 (15.88)
None of the above	16,041 (8.34)	4,465 (9.29)	3,527 (7.34)	3,671 (7.63)	4,378 (9.11)
Missing	818 (0.43)	211 (0.44)	165 (0.34)	200 (0.42)	242 (0.50)
Employed, *n* (%)	179,258 (93.23)	44,626 (92.81)	44,987 (93.64)	44,981 (93.55)	44,664 (92.91)
Alcohol intake, *n* (%)					
Never	6,061 (3.15)	1,573 (3.27)	1,439 (3.00)	1,514 (3.15)	1,535 (3.19)
Previous	5,784 (3.01)	1,459 (3.03)	1,308 (2.72)	1,449 (3.01)	1,568 (3.26)
≤ 3 times a month	39,814 (20.71)	9,587 (19.94)	9,718 (20.23)	9,896 (20.58)	10,613 (22.08)
Once or twice a week	47,824 (24.87)	11,602 (24.13)	12,104 (25.19)	12,093 (25.15)	12,025 (25.01)
≥ 3 times a week	92,697 (48.21)	23,838 (49.58)	23,456 (48.82)	23,100 (48.04)	22,303 (46.39)
Missing	102 (0.05)	23 (0.05)	19 (0.04)	30 (0.06)	30 (0.06)
Smoking status, *n* (%)					
Never	108,682 (56.52)	26,334 (54.77)	28,113 (58.52)	28,100 (58.44)	26,135 (54.36)
Previous	68,115 (35.42)	17,885 (37.20)	16,681 (34.72)	16,380 (34.07)	17,169 (35.71)
Current	15,063 (7.83)	3,759 (7.82)	3,163 (6.58)	3,509 (7.30)	4,632 (9.64)
Missing	422 (0.22)	104 (0.22)	87 (0.18)	93 (0.19)	138 (0.29)
Physical activity (MET-min/week), *n* (%)					
Low	29,500 (15.34)	5,949 (12.37)	6,975 (14.52)	7,882 (16.39)	8,694 (18.08)
Moderate	115,918 (60.29)	28,046 (58.33)	29,622 (61.66)	29,525 (61.41)	28,725 (59.75)
High	46,864 (24.37)	14,087 (29.30)	11,447 (23.83)	10,675 (22.20)	10,655 (22.16)
Diabetes, *n* (%)	8,051 (4.19)	1,802 (3.75)	1,631 (3.39)	1,888 (3.93)	2,730 (5.68)
CVD, *n* (%)	8,273 (4.30)	2,183 (4.54)	1,910 (3.98)	1,888 (3.93)	2,292 (4.77)
Hypertension, *n* (%)	102,342 (53.22)	26,584 (55.29)	25,134 (52.31)	24,799 (51.58)	25,825 (53.72)
Energy intake (kJ/day, mean ± SD)	8,875.65 ± 2,716.68	9,982.98 ± 3,087.94	8,884.73 ± 2,403.85	8,398.31 ± 2,359.80	8,236.48 ± 2,602.94

BMI, body mass index; CVD, cardiovascular disease; MET, metabolic equivalent; SD, standard deviation; TDI, Townsend deprivation index.

*P* values were calculated using analysis of variance and *χ*^2^ test for continuous and categorical variables, respectively. All *P* values were < 0.05.

### Sulfur microbial diet score and CKD risk

**Figure 2:**
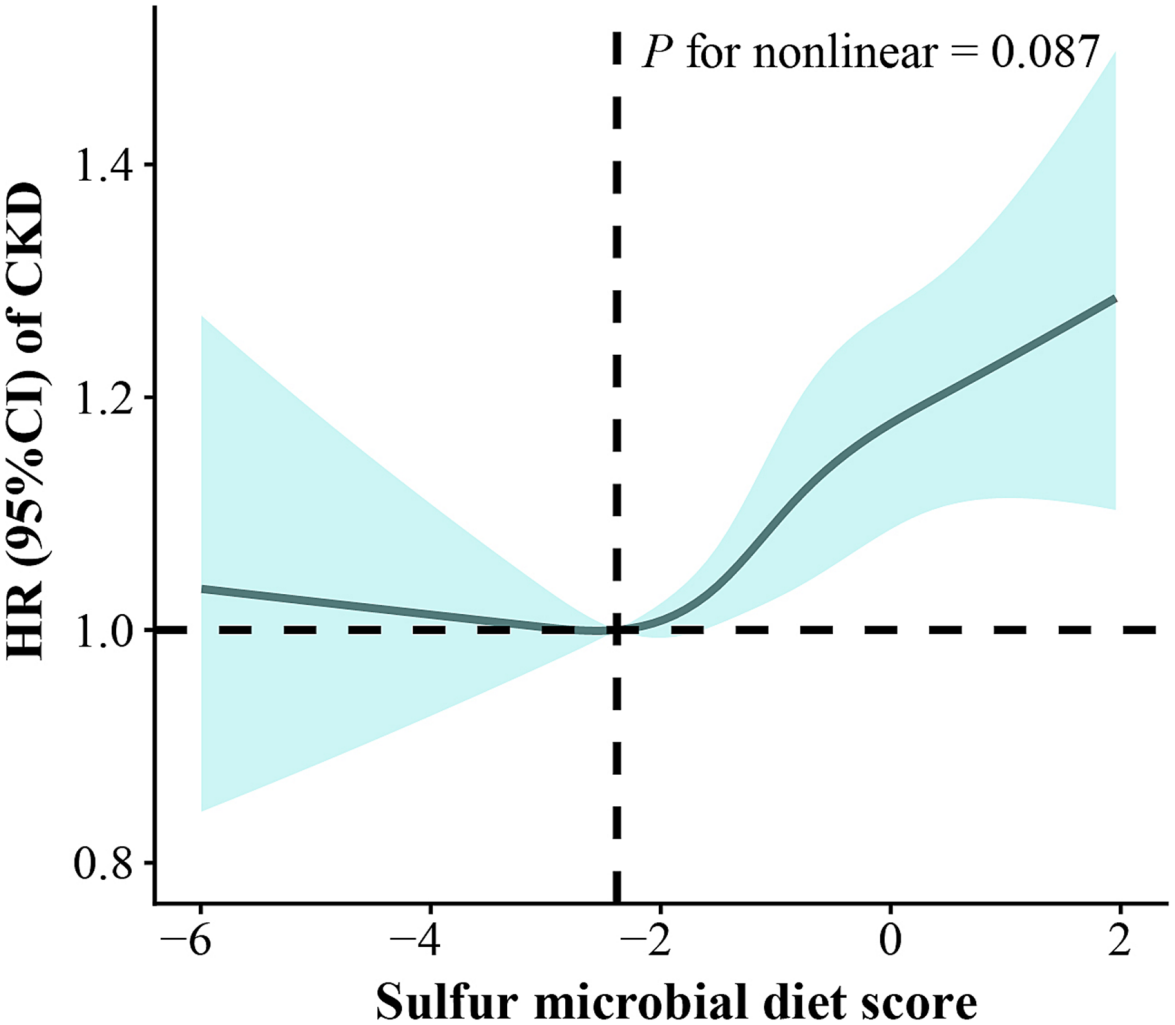
Restricted cubic spline analysis of the relationship between the sulfur microbial diet score and CKD incidence, adjusted for Model 3.

As shown in Table 2, after a median follow-up of 12.17 years (interquartile range: 11.58–12.98 years), we documented 4,739 incident cases of CKD. After adjusting for age, sex, and race, a higher intake of the sulfur microbial diet was significantly associated with an increased risk of CKD. After further adjustment in Model 2, the strength of the association was somewhat attenuated but remained significant. The fully adjusted HR of CKD for participants in the highest quartile was 1.15 (95% CI: 1.06, 1.24) compared with those in the lowest quartile of sulfur microbial diet score. A 6% elevated risk of CKD incidence was observed per SD increase in sulfur microbial diet score. The dose–response relationship, assessed using RCS, was shown in Figure 2 (*P* for nonlinearity = 0.087). Regarding individual components, higher intake of low-calorie drinks, sweets and desserts, as well as a lower intake of other vegetables, were positively associated with the risk of CKD (Supplementary Figure 1).

**Table 2:** Associations between the sulfur microbial diet score and CKD.

CKD	Quartiles of sulfur microbial diet score				per 1-SD	*P*_trend_^1^
	*Q*1	*Q*2	*Q*3	*Q*4		
*N*	48,082	48,044	48,082	48,074		
CKD cases	1,196	1,064	1,145	1,334		
Model 1	1.00 (Ref.)	0.91 (0.84–0.99)	1.02 (0.94–1.11)	1.31 (1.21–1.41)	1.11 (1.08–1.14)	0.259
Model 2	1.00 (Ref.)	0.96 (0.89–1.05)	1.05 (0.97–1.14)	1.15 (1.06–1.24)	1.06 (1.03–1.09)	0.190
Model 3	1.00 (Ref.)	0.96 (0.89–1.05)	1.05 (0.97–1.14)	1.15 (1.06–1.24)	1.06 (1.03–1.09)	0.190

BMI, body mass index; CKD, chronic kidney disease.

Model 1 was adjusted for age, sex and race.

Model 2 was model 1 plus further adjusted for BMI, Townsend deprivation index, educational qualification, household income, employment status, self-reported smoking status, alcohol intake, physical activity level, diabetes, hypertension, and cardiovascular disease.

Model 3 was model 2 plus further adjustment for energy intake.

1 The *P* value for linear trend was calculated using the median of sulfur microbial diet score in each quartile as a continuous variable.

### Substitution analyses

In the substitution analyses (Table 3), replacing one serving per day of low-calorie drinks with other vegetables was associated with a 7% lower risk of incident CKD (HR: 0.93; 95% CI: 0.90, 0.97). Similarly, replacing one daily serving of sweets and desserts with other vegetables was associated with a 5% risk reduction (HR: 0.95; 95% CI: 0.93, 0.97).

**Table 3:** Substitution analyses examining the association between food groups and the risk of CKD.

Substitution analyses	Other vegetables, HR (95%CI)	*P* value	Low-calorie drinks, HR (95%CI)	*P* value	Sweets and desserts, HR (95%CI)	*P* value
With other vegetables	1 (Reference)	NA	0.93 (0.90–0.97)	<0.001	0.95 (0.93–0.97)	<0.001
With low-calorie drinks	1.07 (1.03–1.11)	<0.001	1 (Reference)	NA	1.01 (0.97–1.06)	0.495
With sweets and desserts	1.06 (1.03–1.08)	<0.001	0.99 (0.95–1.03)	0.495	1 (Reference)	NA

BMI, body mass index; CKD, chronic kidney disease.

All models included age, sex, race, BMI, Townsend deprivation index, educational qualification, household income, employment status, self-reported smoking status, alcohol intake, physical activity level, diabetes, hypertension, cardiovascular disease and energy intake.

### Mediation analyses

Mediation analyses revealed that obesity, indicated by BMI and waist circumference, accounted for 23.13% (95% CI: 13.96, 32.30%) and 22.63% (95% CI: 13.23, 32.02%) of the association between the sulfur microbial diet and CKD risk (Table 4). Systemic inflammation, as measured by CRP, partly mediated this association at 1.62% (95% CI: 0.44, 2.79%). Additionally, glucose and HDL cholesterol contributed 2.15% (95% CI: 0.76, 3.55%) and 1.27% (95% CI: 0.17, 2.37%) to the association, respectively. However, no significant mediating effects were observed for triglycerides, total cholesterol, or blood pressure in the relationship between the sulfur microbial diet and the risk of CKD.

**Table 4:** Mediation analyses between the sulfur microbial diet and CKD (per 1-SD).

Potential mediators	Total effect (HR; 95% CI)	*P* value	Direct effect (HR; 95% CI)	*P* value	Natural indirect Effect (HR; 95% CI)	*P* value	Percentage mediated % (95% CI)
Obesity (unadjusted BMI)							
BMI	1.084 (1.051–1.117)	<0.001	1.064 (1.031–1.097)	<0.001	1.018 (1.016–1.021)	<0.001	23.126 (13.955–32.296)
Waist circumference	1.084 (1.049–1.119)	<0.001	1.065 (1.031–1.100)	<0.001	1.018 (1.016–1.020)	<0.001	22.626 (13.230–32.021)
Glucose metabolism (unadjusted DM)							
Glucose	1.064 (1.031–1.097)	<0.001	1.062 (1.029–1.095)	<0.001	1.001 (1.001–1.002)	<0.001	2.154 (0.757–3.551)
Lipid metabolism							
HDL Cholesterol	1.065 (1.031–1.100)	<0.001	1.065 (1.030–1.099)	<0.001	1.001 (1.000–1.001)	0.004	1.271 (0.169–2.373)
Total Cholesterol	1.059 (1.027–1.091)	<0.001	1.059 (1.027–1.091)	<0.001	1.000 (0.999–1.000)	0.926	NA
Triglycerides	1.060 (1.028–1.091)	<0.001	1.059 (1.028–1.091)	<0.001	1.000 (0.999–1.000)	0.631	NA
Blood pressure (unadjusted hypertension)							
SBP	1.055 (1.023–1.087)	<0.001	1.057 (1.024–1.089)	<0.001	0.998 (0.997–0.999)	<0.001	NA
DBP	1.057 (1.026–1.088)	<0.001	1.057 (1.026–1.089)	<0.001	0.999 (0.999–1.000)	0.013	NA
Inflammatory biomarker							
CRP	1.057 (1.026–1.088)	<0.001	1.056 (1.025–1.088)	<0.001	1.001 (1.000–1.001)	<0.001	1.615 (0.439–2.791)

BMI, body mass index; CKD, chronic kidney disease; CRP, C-reactive protein; DBP, diastolic blood pressure; SBP, systolic blood pressure.

All models included age, sex, race, BMI, Townsend deprivation index, educational qualification, household income, employment status, self-reported smoking status, alcohol intake, physical activity level, diabetes, hypertension, cardiovascular disease and energy intake.

### Sensitivity analyses and subgroup analyses

In sensitivity analyses, the results did not materially change when further excluding those experienced events within the first two years of follow-up, those who provided 24-h diet recall data only once, those with extreme energy intake (<3,300 or >18,800 kJ for males or <2,100 or >14,600 kJ for females), those with UACR ≥ 3 mg/mmol, and those with CVD and diabetes at baseline (Supplementary Table 2). Similar results were also observed after additional adjustment for cancer history, medication use, or the healthy diet score, upon recalculating the diet score using Z-standardized servings, or when applying a competing risks model.

**Figure 3:**
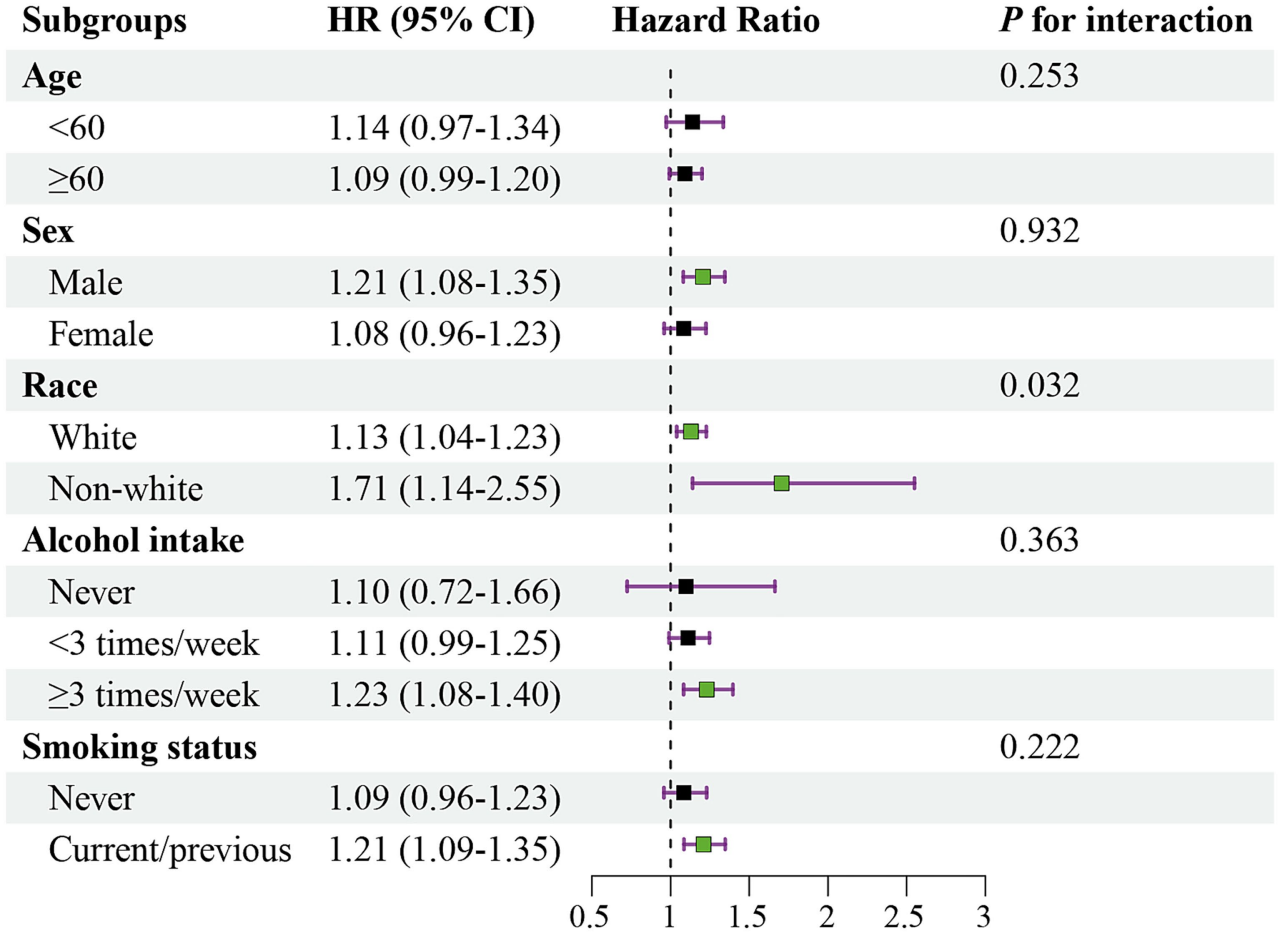
Association of the sulfur microbial diet score with risk of CKD stratified by potential modified factors. Hazard ratios are reported for the highest versus the lowest quartile.

Subgroup analyses were conducted by stratifying participants according to age (<60, ≥60), sex (male, female), race (white, non-white), alcohol intake (never, <3 times/week, ≥3 times/week), and smoking status (never, current/previous). No significant interactions were identified, except for race (Figure 3).

## Discussion

In this large prospective UK cohort study, we observed that a higher sulfur microbial diet score was associated with an elevated risk of CKD. Mediation analyses indicated that obesity, hyperglycemia, HDL cholesterol, and systemic inflammation partially explained this association. Similar results were observed across various sensitivity analyses. In addition, we found that replacing an equal amount of low-calorie drinks or sweets and desserts with other vegetables was significantly associated with a 7% or 5% risk reduction of CKD.

Prior research has primarily examined the relationship between the sulfur microbial diet and gut health outcomes (7, 9). This study comprehensively evaluated the potential adverse effects of sulfur microbial diet intake on kidney function. Our findings indicated that a higher intake of the sulfur microbial diet was associated with an increased risk of CKD. This finding aligned with previous research linking specific dietary components of this diet to CKD risk. For instance, Mirmiran et al. reported that increased consumption of processed meats was associated with an elevated risk of CKD (31), whereas Jhee et al. and Haring et al. found that higher intakes of vegetables, fruits, and legumes were associated with a reduced risk (32, 33).

Diet had a pivotal role in shaping the gut microbiome’s composition, metabolites, function, and diversity (34). Sulfur-metabolizing bacteria could convert dietary sulfur into H_2_S, which has been shown to disrupt the mucus barrier (8). This disruption may facilitate the translocation of gut bacteria-produced toxins, such as indoxyl sulfate, p-cresyl sulfate, and trimethylamine N-oxide, into the systemic circulation. As these toxins are primarily excreted via the kidneys, their accumulation due to impaired excretion could contribute to renal dysfunction (35, 36). Beyond its direct damage to the mucus barrier, H₂S may exacerbate kidney damage by suppressing beneficial cellular functions. *Desulfovibrio desulfuricans*, which was believed to thrive under a sulfur microbial diet, metabolized host-beneficial short-chain fatty acid (SCFAs) such as butyrate and generated H_2_S (37, 38). This compound, in turn, inhibited the cellular oxidation of SCFAs, establishing a detrimental circle of mutually exclusive metabolic interactions that damaged kidney function. Furthermore, sulfur compounds from the diet may influence the relative abundance of these microbes (39). The dysbiosis of the gut microbiome, characterized by a reduction in beneficial symbiotic flora and an overgrowth of pathogenic bacteria, was increasingly recognized as a contributing factor to the onset and progression of CKD (40, 41). Higher intake of the sulfur microbial diet promoted the generation of H_2_S and disrupted the intestinal microbiota homeostasis, thereby jointly facilitating the development, acceleration, and progression of CKD.

In addition to elucidating the association between the sulfur microbial diet and CKD risk, our study also explored the potential pathways linking sulfur microbial diet intake to CKD risk. Mediation analyses suggested that this association was partly explained by metabolic disorders and systemic inflammation. Specifically, obesity measurements, including BMI and waist circumference, mediated 23.13 and 22.63% of this association, respectively. The relationship between the sulfur microbial diet and the risk of obesity has been documented by prior study, with findings indicating a significant association with an elevated risk of obesity (10). Obesity was frequently accompanied by insulin resistance (42), which led to the development of diabetes and dyslipidemia (43, 44). These metabolic derangements were established risk factors for CKD, and obesity would further aggravate these conditions, thereby accelerating the progression of CKD (45, 46). Additionally, adipocytes secrete cytokines and adipokines that promote insulin resistance and induce a chronic low-grade inflammatory state, which could further impair kidney function (47). Just as we discovered in our mediation analysis, CRP also partially mediated the association between the sulfur microbial diet and the risk of CKD. In summary, obesity exacerbated the kidney burden by promoting metabolic disorders, such as insulin resistance and inflammation, thereby enhancing its mediating effect in the association between the sulfur microbial diet and CKD.

To our knowledge, this was the first study to investigate the association between the sulfur microbial diet and the risk of CKD. The large sample size of the UK Biobank, coupled with detailed variable information, further strengthened our study and enabled more comprehensive analyses. Nonetheless, several limitations also need to be considered. Firstly, due to constraints of the UK Biobank data, we were unable to directly assess the participants’ microbiome to validate the sulfur microbial diet score. This limitation may result in the oversight of important variations in gut microbiota that could influence the outcomes. Additionally, the score assumed that all individuals had a comparable microbial composition. However, it is likely that there are personalized responses to the sulfur microbial diet, and potential variability in microbiome composition across individuals. Secondly, while multiple 24-h dietary recalls were conducted at baseline, this method may not accurately reflect a participant’s typical intake and may fail to capture long-term dietary habits. Short-term fluctuations in food choices and intake may obscure the proper relationship between the sulfur microbial diet and the risk of CKD. We recommend that future research employ repeated dietary measurements to capture temporal changes. Thirdly, although we accounted for multiple potential confounders and conducted several sensitivity analyses, the possibility of unmeasured confounders cannot be ruled out, potentially inflating the association between the sulfur microbial diet and CKD risk. Fourth, the mediation analyses in our study assumed a causal relationship. However, the cross-sectional measurement of mediators precluded definitive conclusions about the temporal sequence among the sulfur microbial diet, mediators, and CKD. Future dietary intervention studies are necessary to further validate the proposed mediating pathways in the relationship between the sulfur microbial diet and CKD. Finally, since participants in the UK Biobank were predominantly of White descent and tend to be healthier and of higher socioeconomic status (48), caution was warranted when generalizing our findings to other populations. External validation in more diverse cohorts is essential.

## Conclusion

In summary, this prospective study demonstrated that a higher sulfur microbial diet score was significantly associated with an increased risk of incident CKD, and this association was partly mediated by metabolic disorders and systemic inflammation. Replacing low-calorie drinks or sweets and desserts with other vegetables was linked to a significant reduction in CKD risk. Our study may provide evidence on the role of sulfur-metabolizing bacteria in the association between diet and CKD.

## Data Availability

The original contributions presented in the study are included in the article/Supplementary material, further inquiries can be directed to the corresponding author/s.
