# Supplementary material for: Higher consumption of sulfur microbial diet is associated with the increased risk of chronic kidney disease
Source: Front Nutr. 2026 Jan 13;12:1716058. doi: 10.3389/fnut.2025.1716058 (PMC12845337; doi:10.3389/fnut.2025.1716058)
Supplement: Supplementary file 1 [file Supplementary_file_1.docx]

**Supplementary Table 1.** Diet component definitions used in the sulfur microbial diet.

| **Sulfur microbial diet pattern components with the top factor loadings** | **Representative foods** | **Field IDs** | **β-coefficients (SE)** |
| --- | --- | --- | --- |
| ***Positive associations*** |  |  |  |
| Processed meats | Processed meats, bacon, hot dogs | 103010 (Sausage), 103070 (Bacon), 103080 (Ham) | 0.64 (0.25) |
| Liquor | Vodka, gin | 100730 (Spirits) | 0.31 (0.11) |
| Low-calorie drinks | Low-calorie cola, other low-energy carbonated beverages | 100160 (Low-calorie drinks) | 0.38 (0.11) |
| ***Negative associations*** |  |  |  |
| Beer | Beer | 100710 (Beer) | -0.54 (0.17) |
| Fruit juice | Apple juice or cider, orange juice, grapefruit juice, other fruit juice | 100190 (Orange juice), 100200 (Grapefruit juice) | -0.21 (0.12) |
| Legumes | String beans, peas or lima beans, beans or lentils, tofu or soybeans, alfalfa sprouts | 103270 (Tofu), 104000 (Baked bean), 104010 (Pulses), 104110 (Broad bean), 104120 (Green bean), 104280 (Pea) | -0.64 (0.19) |
| Other vegetables | Celery, mushrooms, green pepper, corn, mixed vegetables, eggplant, summer squash | 104020 (Fried potatoes), 104030 (Baked potatoes), 104050 (Mashed potatoes), 104060 (Mixed vegetable), 104070 (Vegetable pieces), 104080 (Coleslaw), 104090 (Side salad), 104100 (Avocado), 104130 (Beetroot), 104140 (Broccoli), 104150 (Butternut squash), 104160 (Cabbage/kale), 104170 (Carrot), 104180 (Cauliflower), 104190 (Celery), 104200 (Courgette), 104210 (Cucumber), 104220 (Garlic), 104230 (Leek), 104240 (Lettuce), 104250 (Mushroom), 104260 (Onion), 104270 (Parsnip), 104290 (Sweet pepper), 104300 (Spinach), 104310 (Sprouts), 104320 (Sweetcorn), 104330 (Sweet potato), 104340 (Tomato), 104350 (Tinned tomato), 104360 (Turnip), 104370 (Watercress), 104380 (Other vegetables) | -0.30 (0.10) |
| Sweets & desserts | Chocolate bars or pieces, candy bars, cookies, brownies, doughnuts, cake, pie, sweet roll, coffee cake, pastries | 102140 (Milk based pudding), 102150 (Other milk pudding), 102170 (Soya dessert), 102180 (Fruit cake), 102190 (Cake), 102200 (Doughnut), 102210 (Sponge pudding), 102220 (Cheese cake), 102230 (Other dessert), 102260 (Chocolate bar), 102270 (White chocolate), 102280 (Milk chocolate), 102290 (Dark chocolate), 102300 (Chocolate-covered raisin), 102310 (Chocolate sweet), 102320(Diet sweets), 102330 (Sweets), 102340 (Chocolate covered biscuits), 102350 (Chocolate biscuits), 102360 (Sweet biscuits), 102370 (Cereal bar), 102380 (Other sweets) | -0.23 (0.10) |

**Supplementary Table 2.** Sensitivity analyses of the HRs for the associations of the sulfur microbial diet score with CKD.

| **Sensitivity analyses** | **HR (95%CI)** | | | |
| --- | --- | --- | --- | --- |
|  | **Q1** | **Q2** | **Q3** | **Q4** |
| Excluding those who provided 24-h diet recall data only once | 1 (Reference) | 1.01 (0.91-1.13) | 1.07 (0.96-1.19) | 1.18 (1.07-1.32) |
| Excluding those with extreme energy intake | 1 (Reference) | 0.97 (0.89-1.06) | 1.06 (0.97-1.15) | 1.17 (1.08-1.27) |
| Excluding those experienced events within the first 2 years of follow-up | 1 (Reference) | 0.96 (0.88-1.05) | 1.05 (0.97-1.14) | 1.15 (1.07-1.25) |
| Further adjustment for cancer | 1 (Reference) | 0.97 (0.89-1.05) | 1.05 (0.97-1.14) | 1.15 (1.06-1.24) |
| Further adjustment for healthy diet score | 1 (Reference) | 0.95 (0.88-1.04) | 1.03 (0.95-1.12) | 1.12 (1.04-1.21) |
| Excluding those with CVD and diabetes at baseline | 1 (Reference) | 0.93 (0.84-1.02) | 1.03 (0.94-1.14) | 1.12 (1.02-1.22) |
| Excluding those with UACR ≥ 3 mg/mmol at baseline | 1 (Reference) | 0.96 (0.88-1.05) | 1.06 (0.97-1.16) | 1.13 (1.03-1.23) |
| Recalculation of the score using Z-standardized servings | 1 (Reference) | 1.00 (0.92-1.09) | 1.06 (0.98-1.15) | 1.17 (1.08-1.26) |
| Competing risks model | 1 (Reference) | 0.97 (0.89-1.05) | 1.05 (0.97-1.14) | 1.15 (1.06-1.24) |
| Further adjustment for medication use | 1 (Reference) | 0.96 (0.89-1.05) | 1.05 (0.97-1.14) | 1.15 (1.06-1.24) |
| Extreme energy intake: <3300 or >18,800 kJ for males or <2100 or >14,600 kJ for females.  Healthy diet score: Fruits: ≥3 servings/day, Vegetables: ≥3 servings/day, Fish: ≥2 servings/week, Processed meats: ≤1 serving/week, Unprocessed red meats: ≤1.5 servings/week, Whole grains: ≥3 servings/day, Refined grains: ≤1.5 servings/day. The healthy diet score ranged from 0 to 7. Medication use encompassed cholesterol lowering medication, blood pressure medication, and insulin. | | | | |


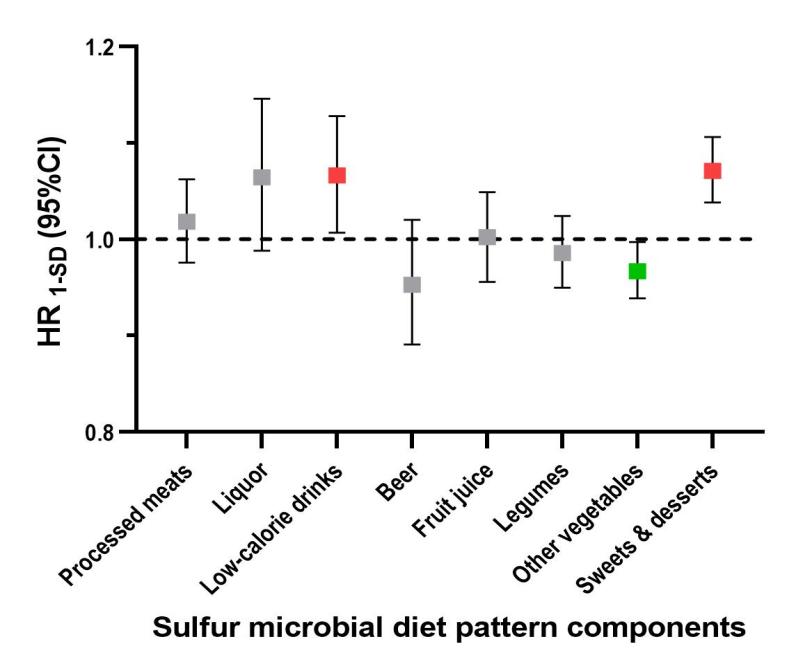


**Supplementary Figure 1.** Associations between specific component of sulfur microbial diet pattern and CKD.

All models included age, sex, race, BMI, Townsend deprivation index, educational qualification, household income, employment status, self-reported smoking status, alcohol intake, physical activity level, diabetes, hypertension, cardiovascular disease and energy intake.
